# Supplementary figures and images for: The Potential Habitat Response of Cyclobalanopsis gilva to Climate Change
Source: Plants (Basel). 2024 Aug 22;13(16):2336. doi: 10.3390/plants13162336 (PMC11360705; doi:10.3390/plants13162336)

avg.diff.AUC

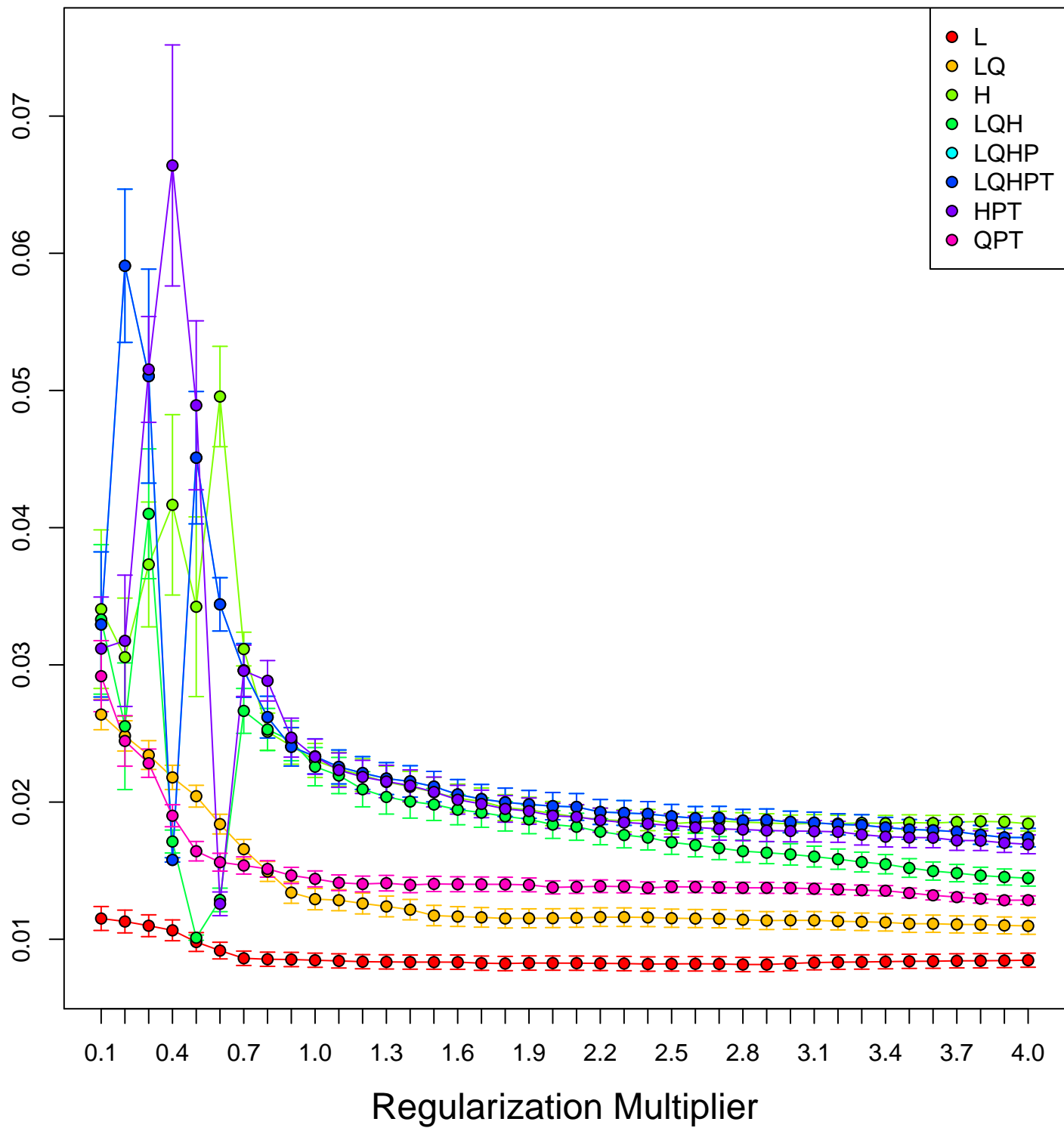

Supplement: Supplementary file 1 [file plants-13-02336-s001.zip › supplementary materials/Figure S1 avg.diff.AUC .pdf]

avg.test.AUC

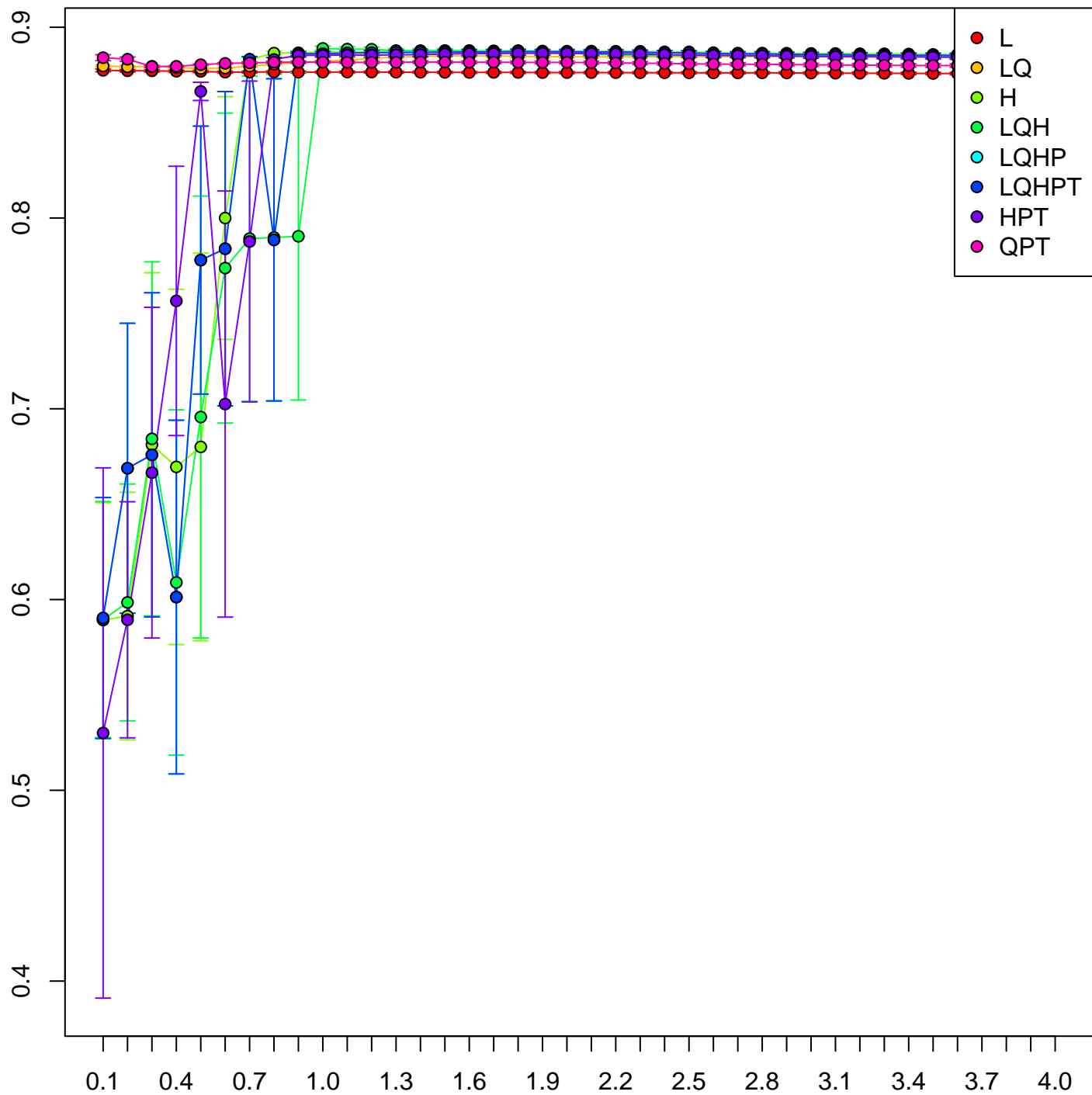

Regularization Multiplier

Supplement: Supplementary file 1 [file plants-13-02336-s001.zip › supplementary materials/Figure S2 avg.test.AUC .pdf]

avg.test.or10pct

0.05  
0.10  
0.15  
0.20  
0.25

0.1 0.4 0.7 1.0 1.3 1.6 1.9 2.2 2.5 2.8 3.1 3.4 3.7 4.0

Regularization Multiplier

- L
- LQ
- H
- LQH
- LQHPT
- HPT
- QPT

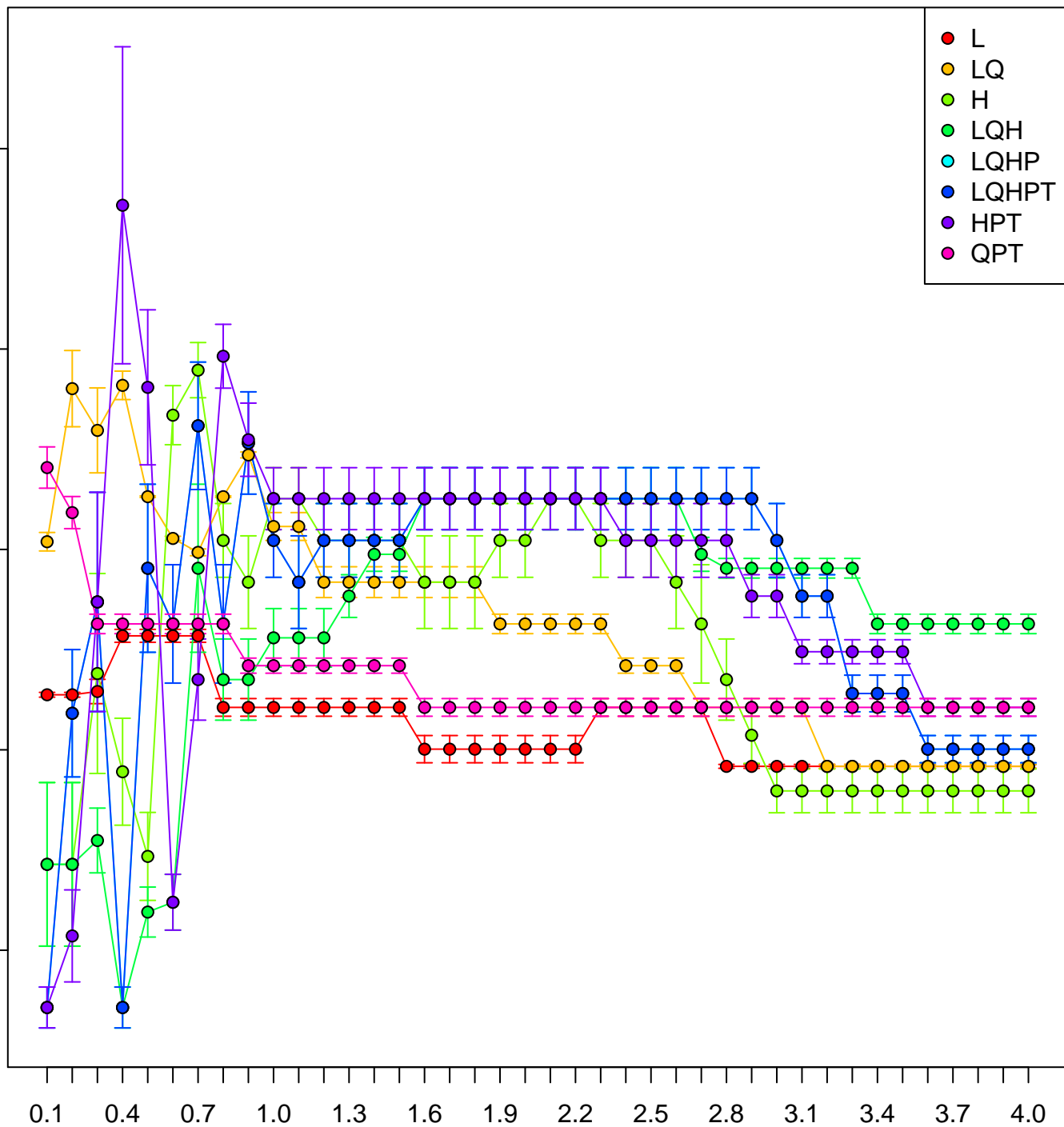

Supplement: Supplementary file 1 [file plants-13-02336-s001.zip › supplementary materials/Figure S3 avg.test.or10pct .pdf]

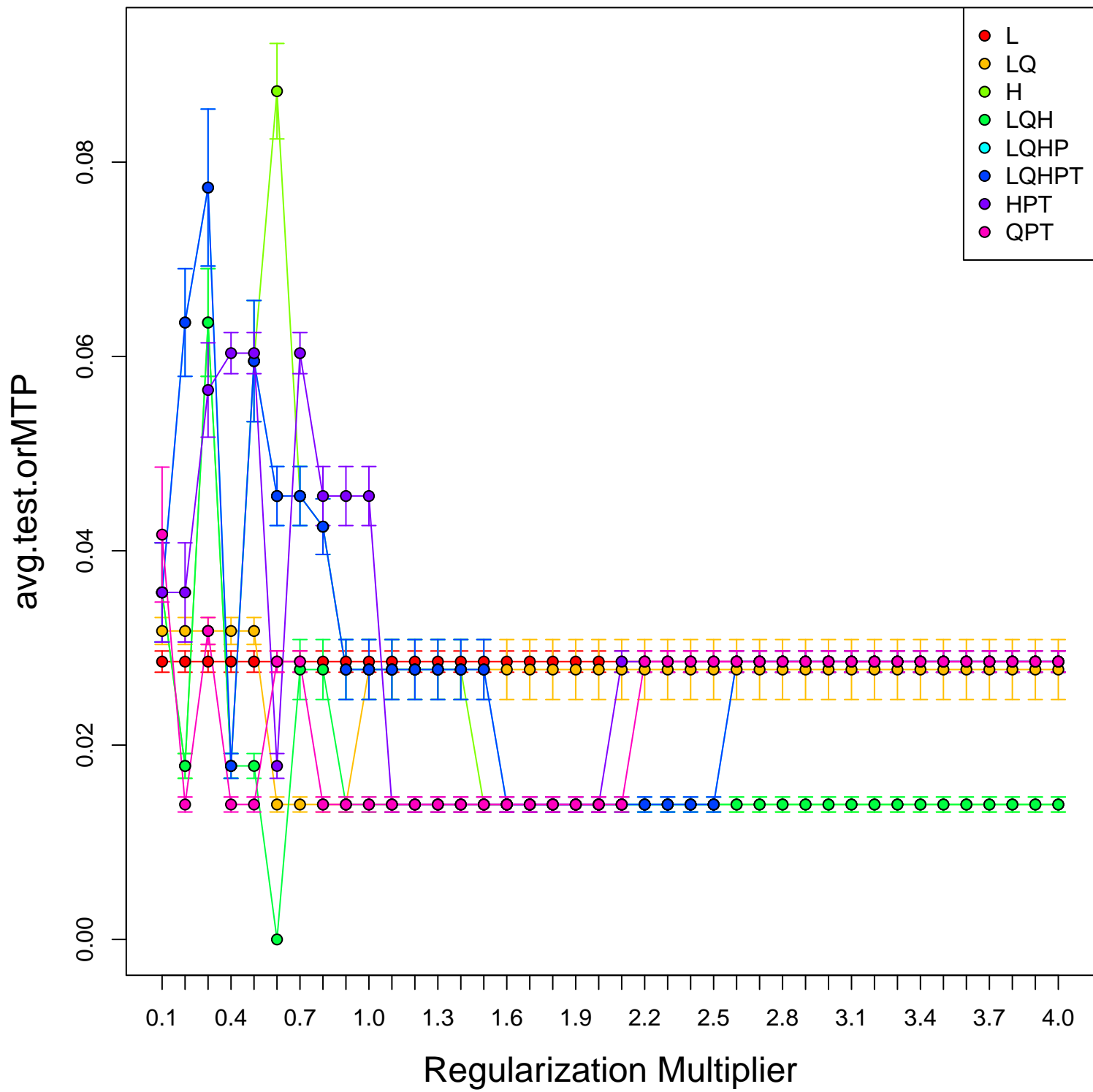

Supplement: Supplementary file 1 [file plants-13-02336-s001.zip › supplementary materials/Figure S4 avg.test.orMTP .pdf]

delta.AICc

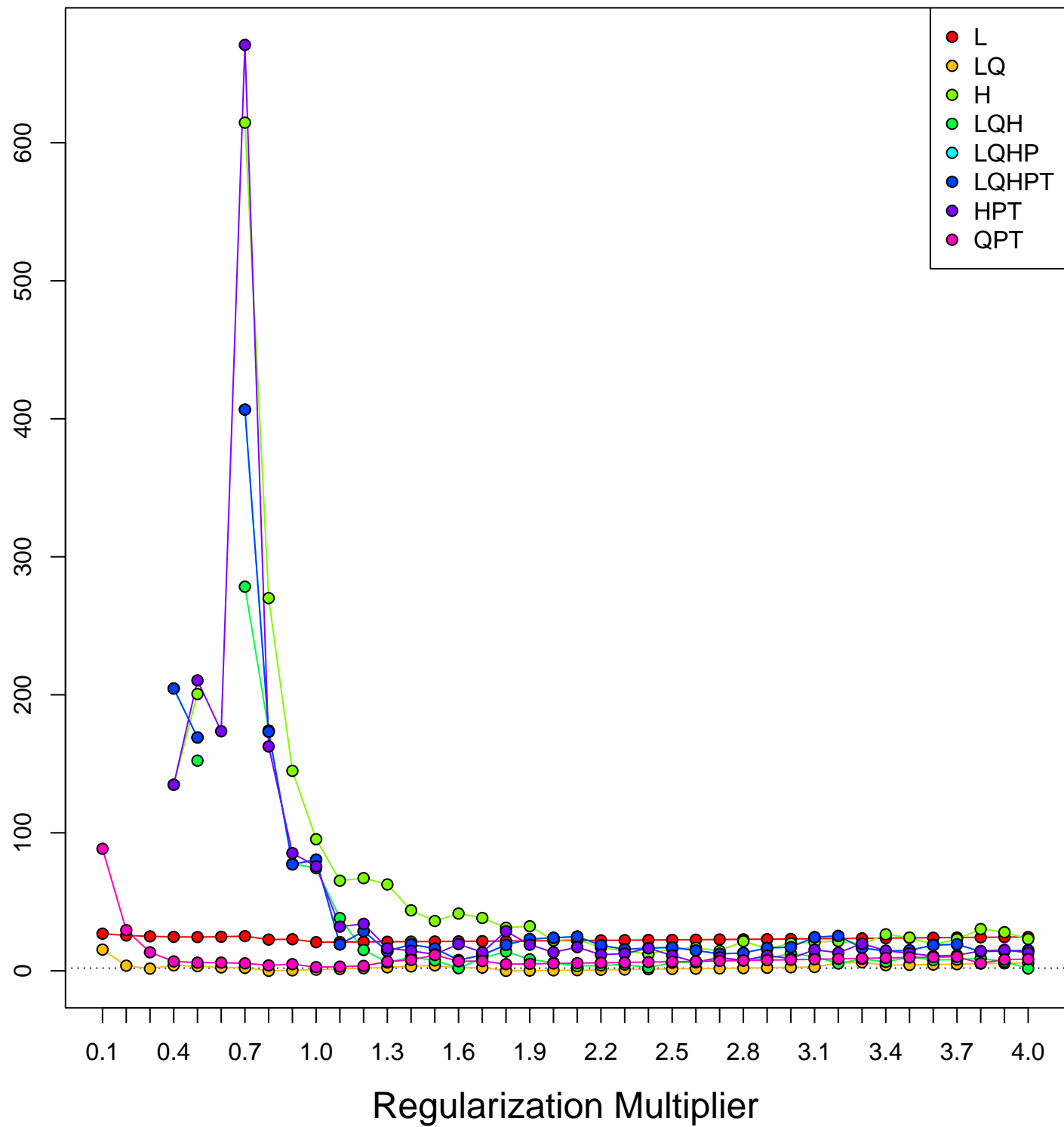

Supplement: Supplementary file 1 [file plants-13-02336-s001.zip › supplementary materials/Figure S5 delta.AICc .pdf]
